# Supplementary figures and images for: Exposure to self-reported traumatic events and probable PTSD in a national sample of Poles: Why does Poland’s PTSD prevalence differ from other national estimates?
Source: PLoS One. 2023 Jul 10;18(7):e0287854. doi: 10.1371/journal.pone.0287854 (PMC10332613; doi:10.1371/journal.pone.0287854)

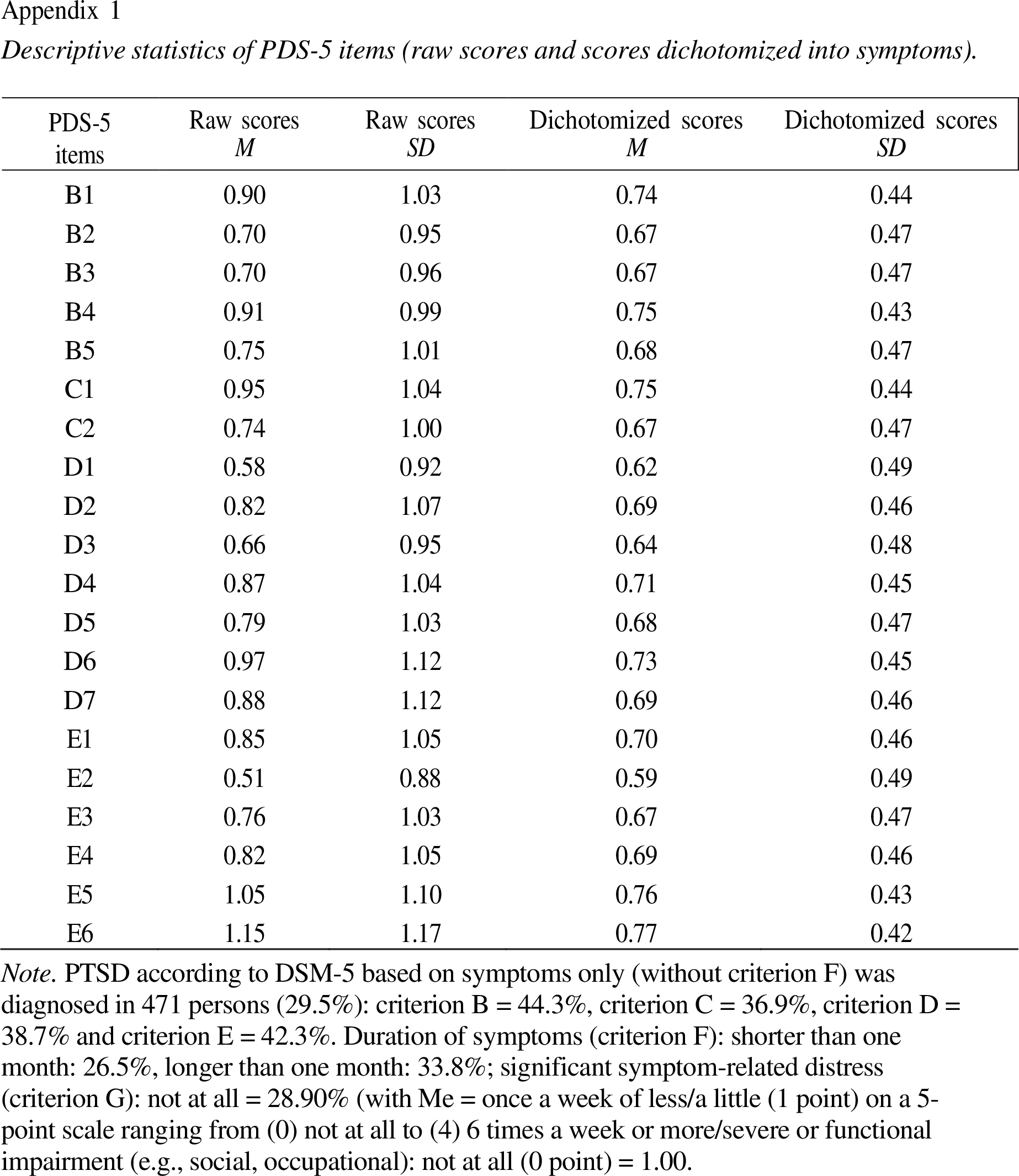

Supplement: S1 Appendix — (TIF) [file pone.0287854.s001.tif]

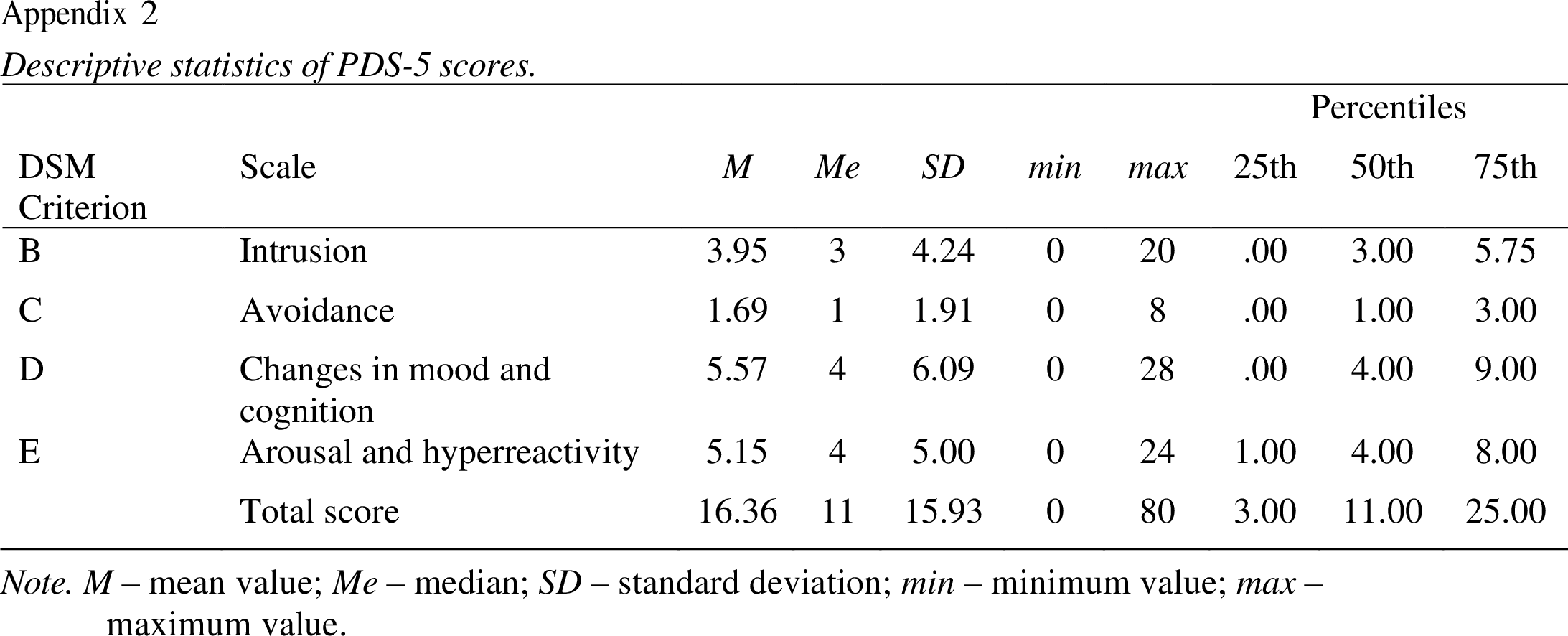

Supplement: S2 Appendix — (TIF) [file pone.0287854.s002.tif]
