# Supplementary material for: Exposure to self-reported traumatic events and probable PTSD in a national sample of Poles: Why does Poland’s PTSD prevalence differ from other national estimates?
Source: PLoS One. 2023 Jul 10;18(7):e0287854. doi: 10.1371/journal.pone.0287854 (PMC10332613; doi:10.1371/journal.pone.0287854)
Supplement: Attachment — Submitted filename: Response to reviewers.docx [file pone.0287854.s004.docx]

**Dear Editor, Dear Reviewers,**

**Thank you very much for your suggestions and remarks concerning our article titled “Exposure to Self-Report Traumatic Events and Probable PTSD in a National Sample of Poles:**

**Why Does Poland’s PTSD Prevalence Differ from Other National Estimates?”, which we would like to publish in *PLOS ONE.* We referred to all reviewers’ remarks. Below we cite every statement and comment of the reviewers and provide their answers in parentheses.**

**Editor’s remarks**

1. Please ensure that your manuscript meets PLOS ONE's style requirements, including those for file naming. The PLOS ONE style templates can be found at [https://journals.plos.org/plosone/s/file?id=wjVg/PLOSOne_formatting_sample_main_body.pdf](about:blank) and
[https://journals.plos.org/plosone/s/file?id=ba62/PLOSOne_formatting_sample_title_authors_affiliations.pdf](about:blank)

**[Thank you for this remark. I can assure that the whole manuscript is in line with PLOS ONE style.]**

2. Please provide additional details regarding ethical approval in the body of your manuscript. In the Methods section, please ensure that you have specified the name of the IRB/ethics committee that approved your study.

**[We have included this additional information in the Method section.]**

3. We note that you have stated that you will provide repository information for your data at acceptance. Should your manuscript be accepted for publication, we will hold it until you provide the relevant accession numbers or DOIs necessary to access your data. If you wish to make changes to your Data Availability statement, please describe these changes in your cover letter and we will update your Data Availability statement to reflect the information you provide

**[Thank you for this information. We have included relevant change of this statement in the cover letter.]**

4. "In your Data Availability statement, you have not specified where the minimal data set underlying the results described in your manuscript can be found. PLOS defines a study's minimal data set as the underlying data used to reach the conclusions drawn in the manuscript and any additional data required to replicate the reported study findings in their entirety. All PLOS journals require that the minimal data set be made fully available. For more information about our data policy, please see [http://journals.plos.org/plosone/s/data-availability](about:blank).

""Upon re-submitting your revised manuscript, please upload your study’s minimal underlying data set as either Supporting Information files or to a stable, public repository and include the relevant URLs, DOIs, or accession numbers within your revised cover letter. For a list of acceptable repositories, please see [http://journals.plos.org/plosone/s/data-availability#loc-recommended-repositories](about:blank). Any potentially identifying patient information must be fully anonymized.

Important: If there are ethical or legal restrictions to sharing your data publicly, please explain these restrictions in detail. Please see our guidelines for more information on what we consider unacceptable restrictions to publicly sharing data: [http://journals.plos.org/plosone/s/data-availability#loc-unacceptable-data-access-restrictions](about:blank). Note that it is not acceptable for the authors to be the sole named individuals responsible for ensuring data access.

We will update your Data Availability statement to reflect the information you provide in your cover letter.

**[Thank you for this remark. We included relevant information about this in the cover letter.]**

The authors need to correct the applied algorithm, because for PTSD two or more symptoms are required for each criteria: D and E. This is a major concern that may affect the reported prevalence of PTSD, title, and discussion.
**[Thank you very much for this remark. We are very grateful for this, as we found that it was some small typo/mistake in the description of these criteria in the Results section. Now in the revised version of the manuscript we modified the results section. This mistake was only present in the description of the results, and not the applied algorithm of the data, which is calculated correctly. In order to be sure, we doubled-checked this issue, and we can confirm that there is no error in the algorithm.]**

Please provide more information about the trauma screening of the PDS-5: How many different types of events were assessed, is there any information of the type of confrontation? How did you ensure that “others” are in line with the definition of traumatic events based on the DSM-5.

**[Thank you very much for this remark. The PDS-5 is a 24-item self-report measure that assesses PTSD symptom severity in the last month according to DSM-5 criteria. An item for each of the 20 DSM-5 PTSD symptoms is included, and an additional four items asking about distress and interference caused by PTSD symptoms as well as onset and duration of symptoms.**

**Symptom items are rated on a 5-point scale of frequency and severity ranging from 0 (Not at all) to 4 (symptom present 6 or more times a week / severe). The types of traumatic events are as following (participants did not include “other” in our sample):**

**- Serious, life-threatening illness (e.g. heart attack)**

**- Assault (armed robbery, serious injury in a fight, someone pointed a gun at you, etc.)**

**- Sexual assault (rape, attempted rape, forced intercourse with e.g. weapons)**

**- Participating in hostilities or staying in an area of such hostilities**

**- Childhood abuse (beating, sexual acts with someone 5 or more years older)**

**- Accident (serious injury or death in a car accident, fire or other domestic accident, etc.)**

**- Natural disaster (strong hurricane, flood, earthquake, etc.)**

**- Other traumatic experience (please briefly describe).**

The paragraph “PTSD Diagnostic Criteria” appears twice in the manuscript and should be removed from the results section.
**[Thank you for this remark. We removed double descriptions of the “PTSD Diagnostic Criteria”.]**

**Reviewer #1:** The paper is Interesting and important in general, not only for Polish readers. It describes the representative prevalence of PTSD and the satisfaction in life which are significant data for any comparative study. I suggest only minor changes in the structure and other in discussion, however depending to the Authors decision.

**[Thank you very much for the nice words on our submission.]**

I suggest to remove the repetition of PTSD diagnostic criteria and put it only in one place.

**[It is very important remark – we removed the repetition of PTSD diagnostic criteria.]**

Authors discuss the high result of the PTSD prevalence in the examined group. In my opinion the easiest explanation is that Authors used DSM-5 criteria but compared with the publications based on more restrictive DSM-IVTR or DSM-IIIR editions.

**[Thank you very much for this remark. However, we cannot agree entirely with this remark, as large epidemiological study conducted by Kilpatrick et al. (2013) showed that the PTSD prevalence estimates in light of DSM-5 are even lower than their PTSD estimates in light of DSM-IV counterparts.**

**Kilpatrick, D. G., Resnick, H. S., Milanak, M. E., Miller, M. W., Keyes, K. M., & Friedman, M. J. (2013). National estimates of exposure to traumatic events and PTSD prevalence using DSM-IV and DSM-5 criteria. Journal of Traumatic Stress, 26(5), 537–547.** [**https://doi.org/10.1002/jts.21848**](about:blank)

**So it is not rather the issue of stringent vs. non-stringent PTSD levels in the subsequent DSM editions, but the case of extraordinary high level of PTSD in Poland, which was found also in the previous studies, yet not conducted in the representative sample as in our current study. ]**

Dragan, M., Lis-Turlejska, M., Popiel, A., Szumiał, S., & Dragan, W. (2012). The validation of the Polish version of the Posttraumatic Diagnostic Scale and its factor structure. European Journal of Psychotraumatology, 3, Article 18479. https://doi.org/10.3402/ejpt.v3i0.18479

Popiel, A., Zawadzki, B., Pragłowska, E., & Teichman, Y. (2015). Prolonged exposure, paroxetine and the combination in the treatment of PTSD following a motor vehicle accident. A randomized clinical trial – The “TRAKT” study. Journal of Behavior Therapy and Experimental Psychiatry, 48, 17–26. https://doi.org/10.1016/j.jbtep.2015.01.002

Discussion is limited to self-quotation but doesn’t mention other researchers as i.e. most known Polish-Jewish Holocaust survivors researcher Maria Orwid.

**[Thank you very much for this remark. We cited the aforementioned researcher in the revised manuscript.]**

In general the correlation between PTSD and less satisfaction in life in groups confirm the results.

**[Thank you for this remark.]**

Reviewer #2: The authors use an established self-report instrument to assess trauma burden, PTSD symptoms and quality of life in a sample representative of the Polish population. The following are the core statements of the article: Both the number of people reporting at least one trauma and the number of self-reported PTSD sufferers are comparatively high The most frequently reported trauma type is experiencing a severe physical illness. The trauma types with the highest likelihood of PTSD are sexual abuse, sexual violence and physical violence. In the PTSD-group young age (18 - 46 years), female gender, low educational status and being single are more prevalent than in the no-PTSD- group. In comparison, the quality of life is significantly lower in the PTSD-group
The study makes an important contribution to identifying the prevalence of PTSD and its specifics in Poland. The representative sample for the Polish population should be emphasized here. In addition, the theoretical and empirical introduction to the research question is very successful and pleasant to read.

**[Thank you very much for the kind words on our submission.]**

There are three major aspects that need to be addressed:
1)The argumentation not to work with the American cut-off of the PDS -5 value is meaningful. At the same time, I find the omission of a quantitative evaluation problematic. A quantitative evaluation would provide us with important information to better interpret the high symptoms recorded in the self-report. The authors do not report how high the scale score must be in order to fulfil one symptom per PTSD criterion. To my knowledge, the PDS -5 counts each item >0. Thus, according to the authors' approach, individuals with a total scale score ≥ 7 would be part of the PTSD-group (comparison: US cut-off score =28). This is difficult against the background of high overlaps of many PTSD symptoms with symptoms of other mental illnesses as well as the fact that it could represent non-clinically relevant manifestations of symptoms. This approach carries the risk of overestimating the prevalence of PTSD. In addition to the evaluation already implemented, a possible approach is to carry out a quantitative evaluation and to present this graphically by means of violin charts or box plots for the total value as well as all subscales.
**[Thank you very much for this remark. However, we feel that there is some misunderstanding. In our study we performed quantitative evaluation of PTSD in light of PDS-5, but not via the cut-off score, but according to DSM-5 criteria. The PDS-5 is a 24-item self-report measure that assesses PTSD symptom severity in the last month according to DSM-5 criteria. An item for each of the 20 DSM-5 PTSD symptoms is included, and an additional four items ask about distress and interference caused by PTSD symptoms as well as onset and duration of symptoms.**

**Symptom items are rated on a 5-point scale of frequency and severity ranging from 0 (Not at all) to 4 (symptoms present 6 or more times a week / severe).**

**In order to clarify the issue of quantitative evaluation we included a table with Posttraumatic Diagnostic Scale for DSM-5 items’ means and SDs for our sample in the Appendix A.**

**We think that the remark of the Reviewer is extremely important in terms of the interpretation of the results. In the future research it would be valuable to look for the overlap of many PTSD symptoms with symptoms of other mental illnesses in the Polish sample.]**

2)With regard to the sample, the following points would be important:
a. For which characteristics is the sample representative?
b. What is their recruitment process? How many of the original people requested did not participate in the study?

**[Thank you very much for this remark. The sample was representative reflecting the characteristics of the Polish population (e.g. gender, age, place of residence, education) according to the data gathered by**

**Main Statistical Office in Poland. (2020)** [**https://demografia.stat.gov.pl/BazaDemografia/StartIntro.aspx**](about:blank) **, and thus followed the standards of conducting representative studies.**

**In particular, the sample of 1,598 participants allowed us to estimate the prevalence rate in the total population of 37,907,704 Poles (see Main Statistical Office 2020) with a maximum error of 1%, assuming the sampling fraction to be equal to 0.1.**

**Finally, the external company, which was responsible for conducting the study and specializes in the nationwide Polish research panels sent the invitation to about 8000 Poles so as to reach the final, representative sample in our particular study of 1598 Poles.]**

3) In the discussion, relevant aspects are well addressed. In my view, one important aspect should be given more attention. How can the excessively high rate of serious physical illness as trauma be explained and the large proportion of the PTSD group.
**[Thank you very much for this remark. It is difficult to directly indicate if the high rate of serious physical illness as trauma can be linked to excessive high PTSD in our sample, however we included appropriate citations on that problem in the manuscript.]**

Furthermore, I see the following ways to improve the manuscript:
In the title you use the word potentially. Here I would suggest speaking of the self-report
**[Thank you very much for this remark. We modified the title according to this suggestion.]**

Please describe your data preparation: Have there been missing values? Have there been outliers? How have you delt with them?
**[Thank you very much for this remark. In the revised version of the Method section we described in more detail the data gathering by the external company. The sample was representative according to the Main Statistical Office in Poland. (2020), including the standards of conducting representative studies (e.g. gender, age, place of residence, education)** [**https://demografia.stat.gov.pl/BazaDemografia/StartIntro.aspx**](about:blank)

**In particular, the sample of 1,598 participants allowed us to estimate the prevalence rate in the total population of 37,907,704 Poles (see Main Statistical Office 2020) with a maximum error of 1%, assuming the sampling fraction to be equal to 0.1.**

**Finally, the external company, which was responsible for conducting the study sent the invitation to about 8000 Poles so as to reach the final, representative sample in our particular study of 1598 Poles.]**

Please describe the psychometric quality of the SWLS and name studies on this subject.
**[Thank you for this remark. We included this information.]**

Please name the use of the Bonferroni correction already in the methods section.

**[We added the comment on the use of Bonferroni correction to the Data Analysis section.]**

Table 1:
Here you name the prevalences in different countries. How did you arrive at the percentages? As you state in the theory section, there are usually several studies with different prevalences. Either you give the range and/or the median in the case of very different values or the mean in the case of similar values. I would recommend including in the notes of the table that it is self-report data.
**[Thank you for this remark. This data was taken from the below references, which we mentioned in the references lists. We also included in the notes of the table that it is self-reported data.**

**Source: Atwoli et al., 2013; Bum et al., 2005; Gaviria et al., 2016; Kilpatrick et al., 2013; Rosenman, et al., 2002; Trautman & Witchers (2018).**

Chart 1:
Please start the Y-axis at zero. The differences between the groups will otherwise be overrepresented.
Again, I would recommend using a graph to map the distribution. Violin charts or boxplots would again be suitable. Alternatively, you could put this in the supplement for the interested reader. In you decide not to integrate it, please plot the standard deviation lines in the chart.
**[We replaced the former figure with boxplots as recommended.]**

Among other things, you repeat the operationalisation of the PTSD diagnosis in the results section. I would suggest avoiding repetitions and only include it in the methods section
**[Yes, it was a typo/mistake – we corrected it.]**

There is a typo on page 4

**[Thank you, we corrected this typo.]**

**To sum up, I would like to thank the Editor and Reviewers again for their time and effort. I found all the comments very useful, and I believe that they helped me to improve the manuscript's quality. I sincerely appreciate the chance you gave me to revise and submit it to be considered for publication in *PLOS ONE.***
