# Supplementary material for: Exposure to self-reported traumatic events and probable PTSD in a national sample of Poles: Why does Poland’s PTSD prevalence differ from other national estimates?
Source: PLoS One. 2023 Jul 10;18(7):e0287854. doi: 10.1371/journal.pone.0287854 (PMC10332613; doi:10.1371/journal.pone.0287854)
Supplement: Attachment — Submitted filename: Response to reviewers vol2.docx [file pone.0287854.s005.docx]

Dear Editor, Dear Reviewers,

Thank you very much for your suggestions and remarks concerning our article titled “Exposure to Self-Reported Traumatic Events and Probable PTSD in a National Sample of Poles: Why Does Poland’s PTSD Prevalence Differ from Other National Estimates?”, which we would like to publish in PLOS ONE. We referred to all reviewers’ remarks. Below we cite every reviewer's statement and comment and provide their answers in parentheses. All the revised parts are highlighted in red font in the revised manuscript.

**Editor’s remarks**
Please include the following items when submitting your revised manuscript:

- A rebuttal letter that responds to each point raised by the academic editor and reviewer(s). You should upload this letter as a separate file labeled 'Response to Reviewers'.
- A marked-up copy of your manuscript that highlights changes to the original version. You should upload this as a separate file labeled 'Revised Manuscript with Track Changes'.
- An unmarked version of your revised paper without tracked changes. You should upload this as a separate file labeled 'Manuscript'.

**[Thank you very much for reminding us about the formal requirements in Plos One. We have included all the aforementioned parts in the second revision of our manuscript.]**

I have noticed that some of the responses were not sufficient to accept the manuscript in its current form. I have highlighted the points that are necessary to accept your manuscript. In response to Reviewer 2's suggestion, please include a visual representation of the distribution of PDS total scores (e.g., histogram, bean plot) in the results section. Furthermore, you should discuss the scoring criteria in light of cut-off values employed in other studies, thereby acknowledging methodological differences and enabling readers to better understand your findings. Specifically, the fact that a total score of ≥ 7 is sufficient to assign individuals to the group of probable PTSD in the current study (using DSM-5 criteria) in contrast to cut-offs of ≥ 28 (in addition to the DSM-5 criteria) used in other samples and countries. This comparison could be made explicit in the discussion sections of the paper, highlighting the methodological difference and potential impact on the comparison of prevalence rates across countries. This could help readers interpret the findings of the current study and understand the potential impact of the scoring criteria on prevalence rates reported across studies. Addressing Reviewer 2's recommendation could help strengthen the methodological rigor of the study and improve the interpretation and generalizability of the findings.

**[Thank you very much for summarizing the Reviewer's 2 remarks. As you may see below, we have introduced all his/her remarks accordingly. However, we would like to draw your attention to one point again. In our study we performed a quantitative evaluation of PTSD in light of PDS-5, but not via the cut-off score, but according to DSM-5 criteria.** **According to the recommendations (see Foa et al., 2016), we decided to use the DSM–5 diagnostic criteria instead of cut-off points because cut-off points tend to be reliable only in the countries in which they were developed. In other words, in the analyzes presented in this article, belonging to the group of people with a probable diagnosis of PTSD was determined on the basis of DSM-5 diagnostic criteria. These criteria correspond to individual items of the PDS-5 questionnaire. We understand the reviewer's remark on this issue and have reflected this in the discussion and limitations section.]**

Please refer to probable PTSD throughout the manuscript.
**[We used this phrase throughout the manuscript.]**
Minors:

Please provide the information about the trauma screening in your manuscript.
**[We provided this information in the Method section, i.e. in the Measures section. The trauma screening was based on the procedure described in the PDS-5 tool used in this study.]**

Please shift the operationalisation for potential PTSD to the methods sections instead of the result section.
**[We moved this information to the methods section.]**

Reviewer #1: I have no comments to the Authors. The paper is interesting and worth of publication. All previous comments have been addressed.

**[Thank you very much for your nice words about our revision.]**

Reviewer #2: The authors have incorporated key points, such as outlining the representativeness of the sample. The main research question remains highly relevant and the results make an important contribution to PTSD research.

**[Thank you very much for the kind words about our submission.]**

However, there is a critical point that they have not adequately addressed, namely the use of a cut-off of ∑=6 and its potential content risks, which may lead to an overestimation of PTSD prevalence. While it is reasonable not to use the US cut-off, the use of such a low cut-off is not conclusive. It should also be noted that a score of 1 on the Likert scale of the PDS-5 means "once a week or less/a little," which means that a person might meet criterion B for example if they think about the incident once a month. As a result, subsyndromal manifestations of the criteria and disorder may not be adequately screened out. Especially since there is considerable overlap between PTSD symptoms and those of other disorders, such as depression. Again, a cut-off that is too low results in a significant overestimation of PTSD prevalence.

**[Thank you very much for these remarks. However, we would like to emphasize one point again. In our study we performed a quantitative evaluation of PTSD in light of PDS-5, but not via the cut-off score, but according to DSM-5 criteria.** **According to the recommendations (see Foa et al., 2016), we decided to use the DSM–5 diagnostic criteria instead of cut-off points because cut-off points tend to be reliable only in the countries in which they were developed. In other words, in the analyzes presented in this article, belonging to the group of people with a probable diagnosis of PTSD was determined on the basis of DSM-5 diagnostic criteria. These criteria correspond to individual items of the PDS-5 questionnaire. We understand the reviewer's remark on this issue and have reflected this in the discussion and limitations section.**

**More specifically, the term "cut-off score" is usually understood as the threshold value of the sum of points in the questionnaire from which (>=) or above which (>) the subjects are included in the analyzed category, in this case the group of people with a probable diagnosis of PTSD. In this sense, the term appears in the responses to the Reviewer's 2 comments. In the analyzes presented in this article, the threshold value was not used at all. Belonging to the group of people with a probable diagnosis of PTSD was determined using DSM-5 diagnostic criteria, which correspond to individual items of the PDS-5 questionnaire.**

**We feel, however, that Reviewer 2's use of the term "cut-off" refers to something else. The Reviewer points out that although individual items of the PDS-5 questionnaire refer to individual DSM-5 diagnostic criteria, the response scale is also used for each of these symptoms:**

**0 = Not at all**

**1 = Once a week or less/a little**

**2 = 2 is 3 times a week/somewhat**

**3 = 4 to 5 times a week/very much**

**4 = 6 or more times a week/severe**

**The Reviewer points out that perhaps if the value of 1 is already considered as an indicator of the occurrence of a single PTSD symptom, it is not enough, because the symptom may occur less than once a week and therefore does not interfere with functioning to such an extent that it justifies making a diagnosis of PTSD on the basis of such rare symptoms. His/her understanding of the term "cut-off" is comprehension at the level of a single item of the questionnaire, but in our study we followed the level of the probable PTSD overall score, as in DSM-5 diagnosis.**

**Therefore, this is a purely substantive issue and requires a diagnosis based on the literature and/or clinical practice. This diagnosis is based on the frequency of occurrence of symptoms that are significant for the subjects' functioning. But this topic is still the subject of ongoing discussion in the literature and there is no consensus in that topic (Foa et al., 2016).**

**And yes, in the discussion of the results, we commented on these issues, as well as the limitations mentioned by the reviewer. Nevertheless, we want to underline the fact that when including the subjects in the group of people diagnosed with probable PTSD, we took into account not only the occurrence of symptoms, but also their duration (criterion F) and - what is important - the impact on the functioning of the subjects (criterion G). In that way our results may be “protected” from overestimation of the level of probable PTSD diagnosis in our sample.**

The authors have provided a table of descriptive statistics at the item level, but the point of criticism raised has not been sufficiently addressed. It would be helpful to include mean values, standard deviations, or median and quartiles, as well as extreme values, depending on the distribution, at both the criterion level and the total score level. This would enable readers to see how many people in the sample have very low values, which could be important for understanding the prevalence of subsyndromal PTSD. In addition, a visual representation by means of a scatter diagram would be even more helpful.

**[Thank you for these remarks. We added Appendix B with descriptive statistics for PDS-5 scores and presented their distributions in Figure 1.]**

**To sum up, on behalf of my co-authors, I would like to take this opportunity to thank the Editor and Reviewers once again for their time and effort. We found all the comments very useful, and I believe they helped us improve the manuscript's quality. I sincerely appreciate the chance you gave us to revise and submit it to PLOS ONE.**
